# Anatomy of the Posterior Extremity

**Published:** 1887-04

**Authors:** Harrison Allen


### II.—A Note on the Anatomy of the Posterior Extremity.

BY HARRISON ALLEN, M. D.

The dissection of the muscles of the hind limb of the elephant was undertaken with a special object in view and a full account of the myology was not intended. Since the elaborate descriptions of Miall and Greenwood (Journ. of Anat. and Physiolog. xii and xiii), little is needed in the way of characterization of the muscles for purposes of identification.

It is believed that the features named in the note may have some value, especially so in the account of the structures of the region of the knee.

The muscle named semimembranosus in Maill and Greenwood's dissection was identified as the semitendinosus. It possesses a high origin from the vertebral elements above the sacrum and a low origin from the tuberosity of the ischium as well as a characteristic attachment to the fold between the semimembranosus and the biceps. In addition to these attachments the muscle secures a second origin from the tuberosity at a point in advance of the common origin of the semimembranosus and the biceps.

Since R. J. Anderson (Journ. of Anat. and Physiol. xvii, 491) reports the sartorius muscle absent, it is only necessary to state in this connection that in the specimen dissected the muscle was present and normally disposed.

The biceps muscle is so closely associated with the gluteus maximus that they are undoubtedly parts of the same general sheet. Cuvier and Laurillard describe the larger superficial part as gluteus maximus. The muscle is closely adherent to the femur by stout fibrous tissue. The fascial extension of the biceps toward the ankle was not as pronounced as in M. and G.'s specimen, and the gastrocnemius was not inserted with it on the calcaneum. But such slight variations are to be expected, and the absence of close connection do not invalidate the conclusions drawn as to the essential genetic relation of these two great muscles.

The popliteus is a well defined muscle for the greater part of its length. It arises from the femur 2½ cm. above the outer condyle by a small rounded tendon which lies about in the middle of the epiphysis. The muscle passes downward and inward and is inserted upon the posterior surface of the tibia after the usual manner. The outer half of the muscle is tendinous its entire length. The fleshy fibres do not appear until the capsule of the joint has been passed. The tendon is free on its outer border but is inseparately incorporated with the prosterior capsule on the inner; so that the tendon and the capsule might be called parts of one and the same structure. In like manner the muscle is directly continous with the external interarticular disc and with the posterior crucial ligament. The outer condyle of the tibia is distinctly grooved to accommodate the tendon.

The belly of the muscle is 10 cm. long. The tendon, 12 cm. long and 2 cm. wide. The width of the line of insertion is 14 cm. The tibial groove is 2 cm. wide.

The articular surfaces of the knee joint are remarkable for the cushion-like character of the cartilages. The average thickness of the cartilage may be said to be about 2 cm. and is three times this thickness on the tibia beneath the tendon of the popliteus muscle. The upper border of the inner condyle is marked with a number of raised terete lines which appear to be formed by the pressure of the capsule. The inner condyle is the broader and the more convex. The outer edges are but slightly molded by the discs, the edge of the inner condyle in this respect being the most marked. The opposed borders of the condyles posteriorly are emarginate for the play of the posterior crucial ligament.

The interarticular discs are both rudimental. The outer is scarcely to be distinguished from the enormously thickened posterior capsule. Neither of the discs make any impression on the contour of the joint or are seen from the sides. The posterior surface of the capsule is composed of loosely fasciculated fibres over the inner condyle, but is much firmer over the outer where it ranges from 1 to 2 cm. in thickness.

The tendon of the gastrocnemius just before reaching the calcaneum crosses the tendon of the plantaris and ends with this muscle on the tendon of the soleus 5 cm. above the calcaneum.

The section of the inner head of the muscle resembles that of the internal pterygoid muscle of man.

The tendon is 1½ cm. wide. The distance from the external malleolus to the line of union of the two heads of the muscle is 21 cm.

The soleus arises from the intermuscular septum, between it and the peroneus brevis, and is fused with the deep flexors for the proximal half of the leg.

The place of union of the tibial and the fibular digital flexors is in the sole at a point opposite the cuboid bone where it is grooved for the peroneus longus. The point also corresponds to the proximal limit of the pad on the sole.

The plantaris arises from the femur at a point so intimately associated with the peroneus longus that the two muscles appear to have a common origin, the width of the tendon is less than 1 cm.

The tibialis posticus runs in a groove on the side of the internal malleolus.

The peroneus longus arises from the femur as is so commonly the case with quadrupeds. It is part of the same mass with the peroneus brevis to a point 32 cm. above the external malleolus. The apparent tendon from the place at which it is free from the muscle to the point at which it turns to gain the sole is $1\frac{2}{3}$ cm. In the sole it follows the rule in lying in the groove in the cuboid bone. The tendon within the muscle can be traced as far as can the tendons of the extensor communis. The muscle sends a slip, which is 2 cm. wide, to the fifth toe.

The peroneus brevis winds round the posterior surface of the external malleolus. The tendon within the muscle can be traced up nearly to each origin. The belly extends 9¼ cm. below the malleolus. The obliquity of the tendon to the axis of the limb is about 30 degrees.

The extensor communis is fleshy for almost its entire length in the lines corresponding to the first, second and third digits, while the slips for the fourth and fifth toes are tendinous.

The slip for the fourth toe separates 7 cm. above the anterior margin of the foot; that for the fifth, 23 cm. Of the two the quintal slip is the more important. This slip is deflected from the main trend of the muscle at a angle of 20 degrees, passes obliquely downward and backward so as to traverse the entire lateral surface of the limb, crosses superficially the tendon of the peroneus brevis 6 cm. above the insertion of this muscle. The tendon can be traced upward to a point 7 cm. from the origin of the muscle.

The extensor brevis digitorum sends a slip to the quintal division of the communis and can be traced the entire length of the muscle.

A second slip serves to strengthen the tendon of the peroneus brevis. A conjugal slip between the extensor brevis and the communis occupies the interval between the tendons of the peroneus brevis and the extensor slip to the fifth toe.

